# The effects of enhanced primary healthcare interventions on primary care providers’ job satisfaction

**DOI:** 10.1186/s12913-020-05183-9

**Published:** 2020-04-15

**Authors:** Wen Jun Wong, Aisyah Mohd Norzi, Swee Hung Ang, Chee Lee Chan, Faeiz Syezri Adzmin Jaafar, Sheamini Sivasampu

**Affiliations:** 1 Centre for Clinical Outcomes Research, Institute for Clinical Research, National Institutes of Health (NIH), Ministry of Health Malaysia, No. 1, Jalan Setia Murni U13/52, Seksyen U13, Bandar Setia Alam, 40170 Shah Alam, Selangor Malaysia; 2 Centre for Clinical Outcomes Research, Institute for Clinical Research, National Institutes of Health (NIH), Ministry of Health Malaysia, No. 1, Jalan Setia Murni U13/52 Seksyen U13, Bandar Setia Alam, 40170 Shah Alam, Selangor Malaysia; 3 Department of Social and Preventive Medicine, Faculty of Medicine, University of Malaya, Kuala Lumpur, Malaysia; 4 Institute for Health Management, National Institutes of Health (NIH), Ministry of Health Malaysia, No. 1, Jalan Setia Murni U13/52, Seksyen U13, Bandar Setia Alam, 40170 Shah Alam, Selangor Malaysia

**Keywords:** Job satisfaction, Primary care, Interventions, Healthcare providers, Evaluation

## Abstract

**Background:**

In response to the rising burden of cardiovascular risk factors, the Malaysian government has implemented Enhanced Primary Healthcare (EnPHC) interventions in July 2017 at public clinic level to improve management and clinical outcomes of type 2 diabetes and hypertensive patients. Healthcare providers (HCPs) play crucial roles in healthcare service delivery and health system reform can influence HCPs’ job satisfaction. However, studies evaluating HCPs’ job satisfaction following primary care transformation remain scarce in low- and middle-income countries. This study aims to evaluate the effects of EnPHC interventions on HCPs’ job satisfaction.

**Methods:**

This is a quasi-experimental study conducted in 20 intervention and 20 matched control clinics. We surveyed all HCPs who were directly involved in patient management. A self-administered questionnaire which included six questions on job satisfaction were assessed on a scale of 1–4 at baseline (April and May 2017) and post-intervention phase (March and April 2019). Unadjusted intervention effect was calculated based on absolute differences in mean scores between intervention and control groups after implementation. Difference-in-differences analysis was used in the multivariable linear regression model and adjusted for providers and clinics characteristics to detect changes in job satisfaction following EnPHC interventions. A negative estimate indicates relative decrease in job satisfaction in the intervention group compared with control group.

**Results:**

A total of 1042 and 1215 HCPs responded at baseline and post-intervention respectively. At post-intervention, the intervention group reported higher level of stress with adjusted differences of − 0.139 (95% CI -0.266,-0.012; *p* = 0.032). Nurses, being the largest workforce in public clinics were the only group experiencing dissatisfaction at post-intervention. In subgroup analysis, nurses from intervention group experienced increase in work stress following EnPHC interventions with adjusted differences of − 0.223 (95% CI -0.419,-0.026; *p* = 0.026). Additionally, the same group were less likely to perceive their profession as well-respected at post-intervention (β = − 0.175; 95% CI -0.331,-0.019; *p* = 0.027).

**Conclusions:**

Our findings suggest that EnPHC interventions had resulted in some untoward effect on HCPs’ job satisfaction. Job dissatisfaction can have detrimental effects on the organisation and healthcare system. Therefore, provider experience and well-being should be considered before introducing healthcare delivery reforms to avoid overburdening of HCPs.

## Background

Job satisfaction is defined as a set of favourable or unfavourable feelings and emotions of employees towards their work [1]. It can be used as an indicator of working-life quality and a reflection of organisational performance [2]. Job satisfaction among healthcare providers (HCPs) has been identified as an important parameter that influences productivity, their commitment to the organisation and patients’ satisfaction [3–5]. Studies have shown that job satisfaction is influenced by several factors including professional accomplishment [6], interpersonal relationship at workplace [7], working conditions, work stress, workload and adequate staffing [8–10]. Additionally, the increasing expectations and growing demands for primary care services to improve healthcare delivery due to the rising burden of non-communicable diseases (NCDs) and multimorbidity has placed additional pressures on HCPs [11].

The prevalence of NCDs is increasing sharply in both low- and middle-income countries (LMICs) including Malaysia in the recent years [12], with cardiovascular diseases (CVD) and diabetes mellitus being the top four leading causes of NCD deaths [13]. The National Health and Morbidity Survey 2015 has shown that approximately two-thirds of Malaysian adults have at least one of the three cardiovascular risk factors: diabetes mellitus (DM), hypertension and hypercholesterolaemia [14]. The Malaysian healthcare system is faced with increasing pressure to deliver quality care to patients with NCDs and their various complications [15]. In order to address the increasing burden of cardiovascular risk factors, the Malaysian government has moved forward to reform primary care delivery system by implementing Enhanced Primary Healthcare (EnPHC) interventions as a demonstration project at community and primary care facility levels. At facility level, EnPHC interventions were delivered as a set of intervention package through person-centred integrated care pathways which aim to improve management and clinical outcomes of type 2 DM (T2DM) and hypertensive patients. A systematic review by Seidu et al. has shown that multicomponent interventions in the primary care settings are more likely to achieve the desired outcomes compared to single intervention. This will be a combination of interventions including audit and feedback, incentivisation, case managers, HCP education and the use of multidisciplinary teams [16].

Numerous studies have shown that changes in the work condition following health system reform can influence HCPs’ job satisfaction and work experiences [17–20]. In fact, adopting new practice models requires changing existing practices and also organisational change which can create undue stress to HCPs [7]. Job dissatisfaction can have detrimental effects on an organisation and a healthcare system where poor job satisfaction is associated with higher level of stress, high staff turnover and poor clinical outcomes [21, 22]. In addition, dissatisfied HCPs may contribute to higher prescribing and medication errors which can jeopardise patient safety [23].

Since primary care professionals serve on the frontline of healthcare, the impact of EnPHC interventions on HCPs job satisfaction serve as an important question in view of growing evidence of dissatisfaction and burnout among healthcare professionals [24, 25]. Although EnPHC interventions were not designed to improve HCPs’ work experience or satisfaction, HCPs’ job satisfaction may improve indirectly via its emphasis on team-based care and increased staff engagement. Conversely, participation in EnPHC interventions can create additional stress to HCPs as clinics were required to change their workflows. The additional tasks introduced by EnPHC interventions may lead to a tremendous strain on the already overburdened and overcrowded public primary care clinics by patients with diabetes, hypertension and hyperlipidaemia [26]. These changes may bring negative impacts on their work experiences which will ultimately lead to higher work dissatisfaction. To date, studies which address job satisfaction of HCPs in Malaysia remain scarce. The majority of studies evaluating job satisfaction following primary care transformation are mainly conducted in developed countries and often lack of a comparison group [18–20, 27]. Therefore, the aim of this study is to evaluate the effects of EnPHC interventions implemented at facility level on HCPs’ job satisfaction in the LMIC context.

### EnPHC interventions

EnPHC interventions have been implemented in 20 clinics since July 2017. The design of these interventions was based on these three elements: i) integrated multi-disciplinary care, ii) continuous improvement of care delivery and iii) improving organisational practices. Table 1 summarises the description of interventions under each element and the HCPs responsible in delivering the interventions.

**Table 1:** Description of EnPHC interventions and responsibilities of healthcare providers at facility level

Elements	Intervention	HCPs responsible
Integrated multi-disciplinary care	• Integrated specialised services (ISS) The role of allied health professionals was strengthened and expanded to ensure multidisciplinary management. Doctors can refer patients for a set of specific and comprehensive health counselling and education services which will be delivered by ISS personnel matching their area of specialisation (e.g. dietary advice and foot care). These services are made more readily available and patients would no longer need to go to secondary or tertiary facilities to acquire these services.	ISS personnel include pharmacists, dietitians, nutritionists, physiotherapists, occupational therapists and medical social workers
	• Cardiovascular care bundle medication therapy adherence counselling Medication therapy adherence counselling services scope was expanded from diabetes-specific to cardiovascular care which can provide more comprehensive counselling targeting CVD risk.	Pharmacists
Continuous improvement of care delivery	• Clinical and prescribing audits New standard clinical and prescribing audits were introduced for quality monitoring and improvement of T2DM and hypertension care processes in the clinics.	Doctors, nurses, Assistant Medical Officers (AMOs), and Care Coordinators
Improving organisational practices	• Primary triage counter This was introduced at the entry point of clinics to decongest the registration counter and effectively direct patients to the respective units based on their healthcare needs so that treatment services can be delivered in a timely manner.	Nurses or AMOs
	• Secondary triage counter Enhancement of CVD risk screening, standardised CVD risk stratification using Framingham risk score and tracing of laboratory results were conducted in secondary triage counter prior to doctor consultation, so that all necessary information is readily at hand during consultation.	Nurses or AMOs
	• Care Coordinators A care coordinator’s role was introduced to improve coordination of activities related to T2DM and hypertension care management including tracing appointment or medication refill defaulters and managing referrals, with the aim of reducing appointment and medication defaulter rates. Care coordinators can obtain appointments for patients who needed specialty referrals by liaising with officer in charge at secondary or tertiary facilities and track patients’ movement through referral process. Care coordinators’ tasks also include monitoring performance of T2DM and hypertension management through audits and facilitating team meetings to discuss on audit findings.	Nurses or AMOs
	• Family Health Team (FHTs) Team-based care was introduced to ensure continuity of care. Each FHT consists of at least a doctor, an AMO, a nurse and a - care coordinator. It functions as a unit to provide healthcare services for patients who resides within the team’s assigned geographical zones.	Doctors, nurses, AMOs and Care Coordinators
	• NCD care form The NCD care form was a new standardised documentation tool designed as a checklist of essential information and processes of care to ensure guideline-adherent T2DM and hypertension management practices and CVD risk monitoring for every clinic visit.	All HCPs
	• Identification of medication refill defaulter Patients with medication possession ratio < 80% were identified by pharmacists and a defaulter list would be given to the care coordinators to facilitate medication refill defaulter tracing.	Pharmacists

*ISS* integrated specialised services, *AMO* assistant medical officer, *HCP* healthcare providers, *NCD* non-communicable diseases, *CVD* cardiovascular diseases, *T2DM* type 2 diabetes mellitus

## Methods

### Study design and setting

The study was part of the EnPHC evaluation project conducted at 40 public primary care clinics. It is a quasi-experimental study with the primary objective of evaluating the impact of EnPHC interventions on processes of care and intermediate clinical outcomes among T2DM and hypertensive patients. The selection of clinics was only from two states in Malaysia - Selangor and Johor given the consideration of budget and capacity to implement EnPHC interventions. The study collected information from three aspects: the clinics (facility survey), HCPs (provider survey) and patients (patient exit survey and retrospective chart review) across 20 intervention and control public clinics matched by the number of family medicine specialists and medical doctors, location of clinics and annual attendance. Each clinic within the pairs was randomly allocated to intervention and control arms. The effect of interventions on HCPs, which is a secondary objective of the evaluation project and the focus of this paper was evaluated using similar study design. Further details on the design and methods of the study have been described in a study protocol which is currently under journal review.

### Study population

Our study included all HCPs who were directly involved in patient management (consisting of family medicine specialists, medical doctors, assistant medical officers (AMOs), nurses, pharmacists, dietitians, nutritionists, physiotherapists, occupational therapists and medical social workers). Medical doctors are licensed doctors with basic medical training whereas AMOs are similar to nurse practitioners in other countries. All HCPs who were present at the study clinics during the data collection period were invited to participate in the survey.

### Data collection timeline

Baseline data collection was conducted between April and May 2017 where a self-administered provider questionnaire was distributed to each eligible HCP during the data collection period. EnPHC interventions were then implemented in July 2017 and were given 3 months to reach full capacity followed by a further 17 months of implementation (October 2017 to February 2019). A post-intervention survey was administered to the HCPs in the same clinics between March and April 2019.

### Survey instrument

Provider questionnaire was developed based on the questions acquired from the General Practitioner (GP) questionnaire in Quality and Cost of Primary Care (QUALICOPC) study [28]. QUALICOPC is a multi-country study which evaluates quality, costs and equity of primary care system in Europe by using four sets of questionnaires. The QUALICOPC General Practitioner questionnaire was adapted and used for Malaysian QUALICOPC study which was conducted between 2015 and 2016. The results of the doctors’ job satisfaction in QUALICOPC Malaysia study have been published [29]. Modification and adaptation of the questions were done for our provider questionnaire and to accommodate for language proficiencies of other healthcare professionals as QUALICOPC General Practitioner questionnaire was initially designed only for doctors.

The English version of the questionnaire was translated to Malay language by two study collaborators who were fluent in both English and Malay. The Malay version of questionnaire was then back-translated into English by two independent translators according to World Health Organization recommendations [30]. Subsequently, the research team compared both versions of questionnaire and resolved any discrepancies to ensure that the translated items retained the same meaning as the original items. Eight HCPs were engaged from two public clinics, which were not part of the study sample, for the questionnaire pre-test. The provider questionnaire collected information on provider demographics and clinics characteristics, workload, quality of care from providers’ perspective, professional roles as well as job satisfaction (Additional File 1 and Additional File 2).

### Dependent variables

Job satisfaction was measured using the following six items: (i) “I feel that some parts of my work do not really make sense”, (ii) “My work still interests me as much as it ever did”, (iii) “My work is overloaded with unnecessary administrative detail”, (iv) “I have too much stress in my current job”, (v) “Being a healthcare provider is a well-respected job”, (vi) “In my work there is a good balance between effort and reward”. All statements were assessed on a 4-point Likert scale: 1 = strongly agree, 2 = agree, 3 = disagree and 4 = strongly disagree. Reverse coding was applied to responses for the following items to keep the scale in the same direction: “work still interesting”, “well-respected job”, “effort-reward balance”. The responses were coded in such a way that a higher score indicates higher job satisfaction (1 = low job satisfaction and 4 = high job satisfaction). For example, a high score on “well-respected job” reflects that the respondent strongly agreed with the statement, which indicates a high job satisfaction. The scores for each item were averaged and subsequently analysed per item.

### Independent variables

Respondent characteristics and other details including age, gender, educational level, overall duration of practice in healthcare, duration of practice in primary care settings, hours spent per week on direct patient care and professional role were collected. The number of hours spent per week on direct patient care was used as a proxy for individual provider workload. The categories for professional role were: doctors (included family medicine specialists and medical doctors), AMOs, nurses and integrated specialised services personnel (pharmacists, dietitians, nutritionists, physiotherapists, occupational therapists as well as medical social workers). In addition, other clinic characteristics relevant for analysis such as location of the clinic (urban/rural) and study arm (intervention/control) were recorded.

### Statistical analysis

Categorical variables were reported in frequencies and percentages while continuous variables were presented as mean and standard deviation (SD). Sociodemographic characteristics of the intervention and control groups were compared using Chi-square and independent *t* tests.

To estimate the effects of EnPHC interventions on HCPs’ job satisfaction, difference-in-differences (DiD) analysis, which is a common method used in quasi-experimental studies was utilised. The impact of the intervention is estimated through the difference between two differences in the outcomes: (i) difference between the pre- and post-intervention periods within control group and (ii) difference between the pre- and post-intervention periods within intervention group [31, 32]. In this study, repeated cross-sectional DiD analysis was conducted separately for each variable of job satisfaction using a multivariable linear regression model (eq. 1) since there were new respondents during post-intervention phase. Hence, the change in job satisfaction following implementation of EnPHC interventions was estimated from DiD analysis after controlling for differences at baseline, HCP and clinic level covariates as the below equation:

1 $$ {Y}_i={\alpha}_0+{\alpha}_1(Intervention)+{\alpha}_2(Time)+{\alpha}_3\left( Intervention\times Time\right)+{\beta}^{\prime }X $$

where *Y*_*i*_ indicates job satisfaction outcomes; *Intervention* = 1 denotes intervention group and zero otherwise; *Time* = 1 indicates post-intervention period and zero otherwise. *β*^′^*X* denotes the linear combination of covariates to be controlled for. The covariates included in each regression model were age, gender, educational level, professional roles, working duration in primary care settings, hours spent per week on direct patient care, and location of clinics (urban/rural). The coefficient of interest, *α*_3_ represents DiD estimate of the intervention effect controlling for *X*. A negative estimate indicates relative decrease in job satisfaction in the intervention group compared with control group.

All models used generalised estimating equations to adjust for clustering of observations within clinics and to estimate change in mean score for job satisfaction using cluster robust standard error (SE). Multicollinearity of the covariates was checked and detected in one pair of covariates; age and overall duration of practice in healthcare. The variable age was deemed more relevant and hence kept in the analysis. A proportion of the HCPs was surveyed only once, at either baseline or the post-intervention phase. Sensitivity analyses were conducted among the subgroup of providers who responded at both baseline and post-intervention phases and these results were compared to the results in the main analysis.

Subgroup analyses were conducted to assess the changes in job satisfaction among all different professional roles using DiD analysis. A *p*-value of < 0.05 was considered as statistically significant. All statistical analyses were performed using “geepack” package in R version 3.5.3 in RStudio version 1.1.463 [33].

## Results

### Respondents characteristics

The response rate of the survey for both baseline and post-intervention was 99.9%. A total of 1042 and 1215 HCPs completed the questionnaire at baseline and post-intervention respectively. Among all respondents in post-intervention phase, more than half (54.4%) also participated in the baseline survey. The mean age of the HCPs between intervention and control groups did not differ at baseline (33.4 vs 33.3 years old) and post-intervention phase (34.1 vs 34.5 years old) (Table 2). The HCPs were predominantly female with nurses making up the largest group, followed by doctors, ISS personnel and AMOs. Nurses reported the longest work experience in primary care. There was no statistically significant difference between intervention and control groups during baseline and post-intervention in all parameters except for the hours spent per week on direct patient care. At baseline, HCPs from clinics in the control group reported spending longer duration on direct patient care (28.8 h vs 27.2 h; *p* < 0.01) as compared to their counterparts in the intervention group.

**Table 2:** Sociodemographic characteristics of healthcare providers

Characteristics	Baseline					17 months				
	Control ***n*** = 498		Intervention ***n*** = 544		***p***-value ^a^	Control ***n*** = 570		Intervention ***n*** = 645		***p***-value ^a^
Age (years) ^b^	33.3	(7.5)	33.4	(7.7)	0.976	34.5	(6.8)	34.1	(7.0)	0.400
Sex = Female	410	(82.3)	455	(83.6)	0.631	477	(83.7)	525	(81.4)	0.331
Categories					0.882					0.507
Doctors	94	(18.9)	104	(19.1)		127	(22.3)	132	(20.5)	
AMOs	63	(12.7)	60	(11.0)		60	(10.5)	75	(11.6)	
Nurses	283	(56.8)	316	(58.1)		309	(54.2)	338	(52.4)	
ISS personnel	58	(11.6)	64	(11.8)		74	(13.0)	100	(15.5)	
Educational level					0.747					0.096
Master Degree	23	(4.6)	20	(3.7)		30	(5.3)	19	(2.9)	
Bachelor Degree	134	(26.9)	148	(27.2)		180	(31.6)	221	(34.3)	
Certificate / Diploma	341	(68.5)	376	(69.1)		360	(63.2)	405	(62.8)	
Overall duration in practice (year) ^b^										
All categories	9.3	(7.1)	9.1	(7.3)	0.658	10.3	(6.5)	10.0	(6.8)	0.430
Doctors	8.0	(5.1)	7.5	(4.9)	0.512	8.4	(4.8)	8.3	(4.8)	0.797
AMOs	7.5	(7.6)	6.7	(7.1)	0.542	8.9	(6.7)	8.9	(6.6)	0.978
Nurses	10.9	(7.6)	10.8	(8.0)	0.831	12.4	(6.8)	12.0	(7.3)	0.578
ISS personnel	5.3	(3.0)	5.4	(4.1)	0.761	5.8	(3.9)	5.9	(4.4)	0.838
Duration in primary care (year) ^b^										
All categories	6.3	(6.3)	6.0	(6.0)	0.471	7.3	(6.0)	7.0	(6.0)	0.395
Doctors	4.7	(4.6)	4.0	(3.8)	0.307	4.6	(4.1)	4.8	(4.3)	0.610
AMOs	5.4	(6.1)	4.7	(6.3)	0.552	6.7	(5.9)	6.2	(5.5)	0.622
Nurses	7.7	(7.0)	7.4	(6.7)	0.615	9.4	(6.3)	9.0	(6.5)	0.362
ISS personnel	3.0	(2.2)	3.5	(2.3)	0.249	3.9	(2.9)	4.1	(3.3)	0.637
Hours spent per week on direct patient care	28.8	(12.2)	27.2	(12.4)	**0.005**	31.4	(9.8)	30.6	(9.5)	0.097
Average consultation length for DM/ HPT/HLD (min) ^b^										
All categories	16.3	(11.4)	16.9	(11.4)	0.513	16.6	(11.1)	17.4	(11.6)	0.348
Doctors	12.3	(7.1)	11.6	(4.5)	0.463	13.0	(6.0)	13.3	(4.9)	0.660
AMOs	12.3	(9.2)	13.2	(9.6)	0.607	12.3	(7.9)	11.1	(5.8)	0.521
Nurses	19.3	(12.2)	20.4	(12.4)	0.472	20.6	(11.4)	19.9	(14.6)	0.678
ISS personnel	19.9	(13.8)	19.2	(13.8)	0.802	19.3	(16.5)	21.7	(12.8)	0.337

*AMO* assistant medical officer, *ISS* integrated specialised service, *DM* diabetes mellitus, *HPT* hypertension, *HLD* hyperlipidaemia

^a^ Chi-square test for categorical and t-test for numerical variable, between intervention and control groups

^b^ Numerical variables are presented in mean (SD), the other variables that are not indicated are presented in n (%)

### Changes in job satisfaction among all HCPs

At post-intervention, HCPs from the intervention group reported higher level of stress with adjusted differences of − 0.139 (95% CI -0.266, − 0.012; *p* = 0.032) (Table 3). The difference was apparent after adjusting for differences in age, gender, educational level, professional roles, duration of practice in primary care settings and duration spent on direct patient care in a week. There were no statistically significant differences between intervention and control groups at the post-intervention phase for the remaining five items. Since there were some HCPs who participated in only one of the baseline or post-intervention phases, a sensitivity analysis was conducted by selecting only HCPs completed the survey in both phases. We found findings which were similar in terms of magnitude, direction and statistical significance with the analysis which included all HCPs (Additional File 3).

**Table 3:** Changes in job satisfaction among all healthcare provider**s** following EnPHC Interventions

Job satisfaction	Intervention Group				Control Group				Difference^*^	Coefficient (SE)	95% CI	*p*-value
	Baseline ***n*** = 544		17 months ***n*** = 645		Baseline ***n*** = 498		17 months ***n*** = 570					
1. Some parts of my work do not really make sense	2.65	(0.79)	2.56	(0.76)	2.60	(0.81)	2.62	(0.78)	−0.11	−0.120 (0.094)	− 0.302, 0.066	0.210
2. My work still interests me as much as it ever did	3.10	(0.58)	3.26	(0.58)	3.10	(0.58)	3.27	(0.60)	- 0.01	−0.001 (0.064)	− 0.127, 0.124	0.980
3. Overloaded with unnecessary administrative detail	2.31	(0.71)	2.38	(0.72)	2.25	(0.74)	2.43	(0.73)	−0.11	− 0.108 (0.071)	− 0.248, 0.032	0.130
4. Too much stress	2.47	(0.74)	2.43	(0.70)	2.45	(0.73)	2.55	(0.68)	−0.14	−0.139 (0.065)	−0.266, − 0.012	**0.032**
5. Well-respected job	3.39	(0.69)	3.33	(0.70)	3.33	(0.66)	3.36	(0.70)	−0.09	−0.083 (0.060)	−0.200, 0.034	0.166
6. Good balance between effort and reward	2.88	(0.69)	2.92	(0.65)	2.92	(0.62)	2.95	(0.65)	0.01	0 (0.078)	−0.153, 0.154	0.995

*HCP* healthcare provider, *CI* confidence interval

^*^Change from baseline to 17 months, intervention group versus control group

Note: Outcomes are adjusted for age, gender, educational level, professional roles, working duration in primary care settings, hours spent per week on direct patient care, location of clinics (urban/rural)

### Changes in job satisfaction by professional roles

Following subgroup analysis, nurses from the intervention group were found to experience increase in work stress after EnPHC interventions with adjusted differences of − 0.223 (95% CI -0.419, − 0.026; *p* = 0.026) (Table 4). In addition, the same group responded that they were less likely to perceive their profession as well-respected following the interventions with adjusted differences of − 0.175 (95% CI -0.331, − 0.019; *p* = 0.027). On the contrary, ISS personnel in intervention clinics were more likely to report a good balance between work and effort with adjusted differences of 0.386 (95% CI 0.033,0.738; *p* = 0.032). On the other hand, there was no significant change seen in any of the job satisfaction items for doctors and AMO groups.

**Table 4:** Changes in job satisfaction by different professional roles (doctors, assistant medical officers, nurses, ISS personnel) following EnPHC interventions

Job satisfaction	Intervention Group				Control Group				Difference^*^	Coefficient (SE)	95% CI	*p*-value
	Baseline		17 months		Baseline		17 months					
Doctors	***n*** **= 104**		***n*** **= 132**		***n*** **= 94**		***n*** **= 127**					
1. Some parts of my work do not really make sense	2.75	(0.81)	2.73	(0.77)	2.69	(0.79)	2.70	(0.76)	−0.03	− 0.056 (0.162)	− 0.374, 0.262	0.728
2. My work still interests me as much as it ever did	3.02	(0.61)	3.20	(0.55)	3.18	(0.57)	3.17	(0.55)	0.17	0.197 (0.102)	−0.003, 0.396	0.053
3. Overloaded with unnecessary administrative detail	2.51	(0.74)	2.55	(0.73)	2.18	(0.76)	2.46	(0.65)	−0.24	−0.290 (0.148)	−0.581, 0.001	0.051
4. Too much stress	2.67	(0.72)	2.74	(0.60)	2.67	(0.63)	2.80	(0.65)	−0.06	−0.077 (0.135)	−0.341, 0.187	0.570
5. Well-respected job	3.04	(0.84)	3.08	(0.74)	3.17	(0.65)	3.17	(0.63)	0.04	0.007 (0.105)	−0.199, 0.214	0.945
6. Good balance between effort and reward	2.63	(0.72)	2.73	(0.68)	2.61	(0.68)	2.76	(0.66)	−0.05	−0.077 (0.146)	−0.364, 0.210	0.599
Assistant medical officers	***n*** **= 60**		***n*** **= 75**		***n*** **= 63**		***n*** **= 60**					
1. Some parts of my work do not really make sense	2.43	(0.77)	2.35	(0.76)	2.54	(0.91)	2.55	(0.81)	−0.09	−0.133 (0.206)	−0.537, 0.271	0.518
2. My work still interests me as much as it ever did	3.10	(0.60)	3.27	(0.60)	3.14	(0.56)	3.40	(0.56)	−0.09	−0.134 (0.148)	−0.425, 0.156	0.364
3. Overloaded with unnecessary administrative detail	2.12	(0.61)	2.28	(0.73)	2.21	(0.83)	2.23	(0.79)	0.14	0.153 (0.175)	−0.191, 0.497	0.383
4. Too much stress	2.30	(0.70)	2.21	(0.62)	2.38	(0.79)	2.35	(0.80)	−0.06	−0.051 (0.172)	−0.388, 0.286	0.770
5. Well-respected job	3.25	(0.75)	3.32	(0.74)	3.30	(0.75)	3.25	(0.93)	0.12	0.122 (0.196)	−0.263, 0.507	0.535
6. Good balance between effort and reward	2.82	(0.68)	3.00	(0.64)	2.92	(0.75)	3.03	(0.80)	0.07	0.105 (0.153)	−0.195, 0.404	0.494
Nurses	***n*** **= 316**		***n*** **= 338**		***n*** **= 283**		***n*** **= 309**					
1. Some parts of my work do not really make sense	2.64	(0.79)	2.47	(0.74)	2.55	(0.79)	2.53	(0.79)	−0.15	−0.154 (0.128)	−0.405, 0.098	0.230
2. My work still interests me as much as it ever did	3.11	(0.58)	3.31	(0.60)	3.10	(0.57)	3.30	(0.61)	0	−0.009 (0.093)	−0.192, 0.174	0.920
3. Overloaded with unnecessary administrative detail	2.29	(0.71)	2.32	(0.71)	2.26	(0.71)	2.42	(0.74)	−0.13	−0.130 (0.105)	−0.337, 0.076	0.217
4. Too much stress	2.42	(0.74)	2.28	(0.73)	2.37	(0.74)	2.45	(0.65)	−0.22	−0.223 (0.100)	−0.419, − 0.026	**0.026**
5. Well-respected job	3.54	(0.59)	3.43	(0.67)	3.41	(0.64)	3.49	(0.68)	−0.19	−0.175 (0.079)	−0.331, − 0.019	**0.027**
6. Good balance between effort and reward	3.03	(0.63)	2.98	(0.62)	3.02	(0.54)	3.06	(0.58)	−0.09	−0.071 (0.099)	−0.265, 0.122	0.468
ISS personnel	***n*** **= 64**		***n*** **= 100**		***n*** **= 58**		***n*** **= 74**					
1. Some parts of my work do not really make sense	2.75	(0.73)	2.83	(0.71)	2.72	(0.79)	2.92	(0.70)	−0.12	−0.073 (0.172)	−0.411, 0.264	0.670
2. My work still interests me as much as it ever did	3.16	(0.57)	3.17	(0.55)	2.95	(0.66)	3.18	(0.67)	−0.22	−0.175 (0.126)	−0.423, 0.073	0.166
3. Overloaded with unnecessary administrative detail	2.28	(0.68)	2.46	(0.67)	2.36	(0.74)	2.53	(0.73)	0.01	0.007 (0.158)	−0.302, 0.316	0.966
4. Too much stress	2.52	(0.71)	2.70	(0.56)	2.53	(0.68)	2.74	(0.64)	−0.03	−0.025 (0.127)	−0.274, 0.223	0.842
5. Well-respected job	3.38	(0.60)	3.33	(0.64)	3.24	(0.63)	3.28	(0.63)	−0.09	−0.075 (0.169)	−0.407, 0.257	0.657
6. Good balance between effort and reward	2.63	(0.75)	2.89	(0.68)	2.90	(0.58)	2.81	(0.70)	0.35	0.386 (0.180)	0.033, 0.738	**0.032**

*ISS* integrated specialised service, *CI* confidence interval

*Change from baseline to 17 months, intervention group versus control group

Note: Outcomes are adjusted for age, gender, educational level, professional roles, working duration in primary care settings, hours spent per week on direct patient care, location of clinics (urban/rural)

## Discussion

To the best of our knowledge, this is the first study conducted in LMIC which assess the differences in job satisfaction among HCPs following primary care transformation in public primary clinics. These interventions introduced changes in clinic workflow and delegation of new responsibilities to AMOs and nurses in order to achieve greater coordination and continuity of care.

Following implementation of EnPHC interventions, our study demonstrates that HCPs from intervention group were more likely to report of having too much stress compared to their counterparts in control group. These changes were attributed to nurses being the largest group of HCPs in the primary care clinics which was demonstrated in the subgroup analysis. These findings may reflect the growing workload on HCPs following initiation of EnPHC interventions. HCPs were expected to adopt the new interventions in managing patients with T2DM and hypertension and new tasks including audits were added to ongoing duties with little additional resources [34] . All these new changes in the workplace confer higher stress levels to HCPs. Similarly, studies conducted in the United States of America [19, 20] indicated that HCPs perceived their jobs to be more stressful following interventions. This may indicate the presence of change fatigue whereby an individual perceived that too much change is taking place [35]. Whilst this 17-month intervention period can be seen as sufficient time for adaptation, change fatigue is still a possible consequence because new staff are gradually recruited during this period to cope with increasing workload and this can have negative implications such as [36] exhaustion, burnout and high turnover intentions [35, 37]. Moreover, it can potentially jeopardise team commitment and quality of patient care [35, 38, 39]. In addition, we postulate that the slight increase in score among control group could be due to low staff turnover comparing to intervention group. Due to a possible lower turnover rate, HCPs in control group would have better interpersonal relationship at workplace and they will be getting more familiar with their job scopes and working conditions. Hence, they might be more satisfied with their jobs in the long run.

Our findings also showed that nurses are more dissatisfied following implementation of EnPHC interventions compared to other healthcare professionals. The nursing profession has been identified by a number of studies as a stressful occupation [40–42] since they provide not only care to patients and assistance to doctors but also to patients and their family members in terms of health education. Often than not, nurses have to juggle multiple tasks at the same time and tend to be undervalued. During the EnPHC interventions period, nurses were involved in seven out of nine interventions listed in Table 1. It was noted that secondary triage counter was mainly manned by nurses and a significant bulk of EnPHC interventions took place there. Hence, nurses would have to carry out most of the procedures such as foot examinations for diabetic patients, measuring vital signs and BMI, performing CVD risk screening using Framingham risk score, electrocardiography and venepuncture when required. Based on unpublished results from a facility survey in EnPHC evaluation project, there was a 4.5% decline in the number of nurses in intervention clinics as opposed to the increased number of doctors, AMOs and ISS personnel seen. As a result, there may be an increase in workload for nurses due to staff shortage. Changes in the workplace and increased workload can contribute to additional stress due to the additional interventions that need to be conducted. The stressful conditions may lead to a burnout which is a stress-related affective disorder as characterised by emotional exhaustion, depersonalisation and decreased sense of personal achievement [43, 44]. Similarly, the decreased in nurses’ perception towards their profession may be contributed by the lack in public recognition, low salary, poor working conditions [45, 46] and feeling undervalued at work. Indeed, our findings highlight the potential unintended effects of participation in complex interventions, which can magnify dissatisfaction among primary care nurses who are already at high risk of getting burnout [47].

On the contrary, EnPHC interventions tend to have a positive impact on ISS personnel who were more likely to report a good balance between effort and reward. It is plausible that higher level of teamwork and engagement with patients and other healthcare providers contribute to this effect. For example, pharmacists have been entrusted to assist care coordinators in medication refill defaulter tracings apart from the provision of medications to improve medication adherence in T2DM and hypertensive patients. In addition, the expansion of medication treatment adherence clinic services’ scope from diabetes to the overall cardiovascular risk management enables utilisation of their skills and knowledge in a wider and a more diverse group of patients [34]. It is postulated that improved coordination for ISS referral and additional services provided to patients may increase their sense of purpose, team spirit and participation in multidisciplinary management.

### Implications for practice, policy and research

It is undeniable that healthcare professionals play crucial roles in healthcare service delivery and provision of quality care to patients. In recent years, primary care reform has become an imperative for health system worldwide due to the rising burden of non-communicable diseases, aging population and multimorbidity [48, 49]. Unfortunately, these changes and added pressure resulting from transformation initiatives have negative consequences on HCPs [25]. Therefore, understanding the impact of EnPHC interventions on HCPs’ job satisfaction has a clear policy relevance to help policymakers and healthcare leaders in addressing any shortcomings and improving work conditions for HCPs following healthcare reform. Obtaining this information can provide insights and information to policymakers before moving to scale up the EnPHC interventions nationwide. In order to optimise healthcare system performance, new healthcare designs should be developed to achieve the quadruple aim of healthcare system: improving population health, enhancing patient experience, reducing healthcare costs and improving the work life of HCPs [50]. Thus, policymakers need to consider the wellbeing of HCPs while seeking to transform healthcare delivery. Qualitative studies may be proposed to explore the source of dissatisfaction among HCPs which can be attributed to the intervention content or delivery and effectiveness with which the intervention is implemented. Further research is needed to identify optimal approaches for implementation of these interventions to avoid overburdening of HCPs.

## Strengths and limitations

Our quasi-experimental study design with presence of a comparison group is considered a strong observational study design. Another strength of this analysis is the high response rate of HCPs in this evaluation and this can be attributed to the endorsement of this project by the Family Health and Planning Division, Ministry of Health and the fact that all HCPs surveyed were employed within the public health sector. Other strengths of this study include its large sample size and measurement of baseline differences between both groups which enables detection of longitudinal effects of EnPHC interventions on HCPs. One of the limitations of this study is that we were unable to test for parallel trends in the pre-intervention period, a key assumption to DiD analysis. This is because data on trends prior to intervention was not available. Hence, the intervention effects on HCPs job satisfaction should be interpreted with caution and may not be concluded as direct causal link of the findings. Also, the findings are only generalisable to other Malaysian public primary clinics which share similar characteristics. Another limitation is that we were unable to distinguish which interventions led to the dissatisfaction among the HCPs given that all interventions were carried out concurrently.

## Conclusions

Implementation of EnPHC interventions creates both positive and negative impacts on the job satisfaction of HCPs which vary by their professional roles and additional tasks to be conducted. Following implementation, HCPs which provide specialised services such as pharmacists and nutritionists reported higher job satisfaction while nurses conversely reported higher stress levels and being “under-respected”. Our study highlights the importance of evaluating the impact of introducing healthcare delivery reforms on the job satisfaction of HCPs. Therefore, in order to optimise healthcare system performance, provider experience and well-being should be considered when designing health interventions.

## Supplementary information

**Additional file 1.**

**Additional file 2.**

**Additional file 3: Table S1.** Changes in job satisfaction among cohort group following EnPHC Interventions.

**Additional file 4.**

**Additional file 5.**

## Data Availability

The datasets used for this study are available from the corresponding author on reasonable request.

## Abbreviations

EnPHC: Enhanced Primary Healthcare
NCD: Non-communicable diseases
LMICs: Low- and middle-income countries
T2DM: Type 2 diabetes mellitus
HCP: Healthcare provider
AMO: Assistant medical officer
QUALICOPC: Quality and Cost of Primary Care
ISS: Integrated specialised services
SD: Standard deviation
DiD: Difference-in-differences
SE: Standard error
