# Supplementary material for: The effects of enhanced primary healthcare interventions on primary care providers’ job satisfaction
Source: BMC Health Serv Res. 2020 Apr 15;20:311. doi: 10.1186/s12913-020-05183-9 (PMC7158075; doi:10.1186/s12913-020-05183-9)
Supplement: Supplementary file 1 — Additional file 1. [file 12913_2020_5183_MOESM1_ESM.pdf]

THIS QUESTIONNAIRE IS ONLY FOR FAMILY MEDICINE SPECIALISTS, OTHER CLINICAL SPECIALISTS, MEDICAL OFFICERS, HOUSE OFFICERS, NURSES AND ASSISTANT MEDICAL OFFICERS

EVALUATION OF ENHANCED PRIMARY HEALTHCARE (EnPHC) PROVIDERS QUESTIONNAIRE

Instruction:
Where check boxes ☐ are provided, check (√) one or more boxes.
Where radio buttons ◯ are provided, check (√) one box only.

Name of KK: ______________________________

Date of survey conducted: _______________________

This entry belongs to:
○ Cross-sectional (Baseline)
○ Cross-sectional (Post-intervention)

EnPHC Provider ID:-

_____ _____ _____

No	Question	Response categories
	**(A) PROVIDER DEMOGRAPHICS**	
1.	Sex	◯ Male<br>◯ Female
2.	Date of birth	_ _ / _ _ / _ _ _ _<br>(dd / mm / yyyy)
3.	Level of education	◯ Secondary Education (e.g. SPM/SRP)<br>◯ Certificate/Diploma<br>◯ Bachelor Degree<br>◯ Master Degree<br>◯ Other Specify: _________________
4.	Please select one of the following discipline categories	◯ Family Medicine Specialist (FMS)<br>◯ Other Clinical Specialist<br>◯ Medical Officer In Charge (MOIC)<br>◯ Medical Officer<br>◯ House Officer<br>◯ Assistant Medical Officer (AMO)<br>◯ Registered Nurse<br>☐ Staff nurse<br>☐ Chronic Disease Nurse<br>☐ Diabetic nurse<br>☐ Community/Home Care Nurse<br>☐ MCH Nurse<br>☐ TB Nurse<br>◯ Others, specify: ____________________

THIS QUESTIONNAIRE IS ONLY FOR FAMILY MEDICINE SPECIALISTS, OTHER CLINICAL SPECIALISTS, MEDICAL OFFICERS, HOUSE OFFICERS, NURSES AND ASSISTANT MEDICAL OFFICERS

5.	Are you a permanent staff in this KK?	◯ Yes<br>◯ No<br><br>If No, Specify which is your permanent KK/Facility<br><br>______________________________	
6.	Are you currently a visiting staff in other KK(s)?	◯ Yes<br><br>If yes, please specify the KK(s) you visit. Please rank in order from highest workload (1) to lowest workload (10)<br><br>1. KLINIK<br>2. KLINIK<br>3. KLINIK<br>4. KLINIK<br>5. KLINIK<br>6. KLINIK<br>7. KLINIK<br>8. KLINIK<br>9. KLINIK<br>10. KLINIK<br><br>◯ No	
7.	Duration in service	_ _ Years _ _ Months	
8.	Duration in primary care (KK, KKIA, KD, K1M & KK UTC)	_ _ Years _ _ Months	
9.	What is your role in this clinic?<br>(more than one answer is possible)	☐ Part of the Family Doctor Concept (FDC) / Family Health Care Team (FHT)<br>☐ Care Coordinator (CC)<br>☐ Triage staff<br>☐ MCH<br>☐ None of the above	Tasks of CC include:<br>-Ensure relevant tests/examinations were done on time.<br>-Trace defaulters (visit and medication defaulters)<br>-Monitor performance of NCD management and target (audit)<br>-Coordinate patients' referral and counter referral

THIS QUESTIONNAIRE IS ONLY FOR FAMILY MEDICINE SPECIALISTS, OTHER CLINICAL SPECIALISTS, MEDICAL OFFICERS, HOUSE OFFICERS, NURSES AND ASSISTANT MEDICAL OFFICERS

**(B) WORKLOAD**			
10.	How many hours do you work per week? (exclude out-of-hours service/ on-call/ additional jobs/locum)	**38.5** hours per week	
11.	How many of these hours do you spend on direct patient care? (including consultation, home visits, telephone consultations Excluding administrative work, meetings, CME)	_ _ hours per week	
12.	How many patient contacts do you have on a normal working **day**?<br>(Including all patient contact in all your visiting clinic(s) if you are a visiting staff)		
12a.	Face to face	_ _ patients	
12b.	By Telephone	_ _ patients	
13.	On average, how long does a regular diabetes/hypertension/hyperlipidaemia **consultation** usually take?	_____minutes<br>◯ Not applicable	
14.	In a normal working **week**, how many patients do you see:		
14a.	At home visits	_ _ _ patients	
14b.	In other health facility or settings (e.g. school/prison/orphanages)	_ _ _ patients	
14c.	In community-based programs (e.g. KOSPEN)	_ _ _ patients	
15.	In the **past working month** (excluding holidays etc), how often and for how long did you have on-call duties during evenings, nights and weekends?<br><br>* Only include <u>time spent attending cases</u> during the passive on-call period and extended hours<br><br>For the question of evening/night/weekend, the calculation for "How many times" would be how many times you were called to the clinic and the "total" is the total hour(s) spent working in the clinic during that passive on-call period.<br><br>Example,<br>Your passive on-call period in February was for 2 consecutive weeks. If you were called to the clinic 3 times during passive on-call period and no extended hours work.<br>Answer: How many times: 3<br>Total: (no. of hours spent – 1st time) +<br>(no. of hours spent – 2nd time) +<br>(no. of hours spent – 3rd time)	During evening(s) (5pm - 10pm)	How many times: _ _<br>Total: _ _ _ hours
		During night(s) (10pm - 8am)	How many times: _ _<br>Total: _ _ _ hours
		During weekend	How many times: _ _<br>Total: _ _ _ hours

THIS QUESTIONNAIRE IS ONLY FOR FAMILY MEDICINE SPECIALISTS, OTHER CLINICAL SPECIALISTS, MEDICAL OFFICERS, HOUSE OFFICERS, NURSES AND ASSISTANT MEDICAL OFFICERS

(C) QUALITY OF CARE			
**No**	**Question**	**Response categories**	
16.	Do you use clinical guidelines in the treatment of the following:		
16a.	Diabetes Mellitus	◯ Yes<br>◯ No	If yes, please specify the guidelines used:<br>□ MOH CPG Management of T2DM, 5th Edition<br>□ MOH CPG Management of T2DM, 4th Edition<br>□ NICE Guideline T2DM in Adults: Management<br>□ ADA Standards of Medical Care in Diabetes<br>□ AACE/ACE Comprehensive T2DM Management Algorithm<br>□ AACE/ACE DM Guidelines<br>□ Others, please specify(please attach)_______________
16b.	Hypertension	◯ Yes<br>◯ No	If yes, please specify the guidelines used:<br>□ MOH CPG Management of Hypertension, 4th Edition<br>□ MOH CPG Management of Hypertension, 3rd Edition<br>□ JNC 8 Guidelines for the Management of Hypertension<br>□ AACE/ACE Hypertension Guideline<br>□ National Heart Foundation of Australia: Guideline for the Diagnosis and Management of Hypertension in Adults<br>□ ESH/ESC Guidelines for the Management of Arterial Hypertension<br>□ Others, please specify(please attach)_______________
16c.	Dyslipidaemia	◯ Yes<br>◯ No	If yes, please specify the guidelines used:<br>□ MOH CPG Management of Dyslipidemia, 5th edition<br>□ MOH CPG Management of Dyslipidemia, 4th edition<br>□ MOH CPG Management of Dyslipidemia, 3rd edition<br>□ AACE Management of Dyslipidemia and Prevention of CVD<br>□ ESC CPG Management of Dyslipidemia<br>□ Others, please specify(please attach)_______________
17.	In the past 12 months, have you been involved in a disease management programme for patients with the following chronic conditions? (such programmes are multidisciplinary approaches across practices, often based on protocols.)<br><br>Diabetes Mellitus<br>Hypertension<br>Dyslipidaemia	 <br><br><br>◯ Yes ◯ No<br>◯ Yes ◯ No<br>◯ Yes ◯ No	

THIS QUESTIONNAIRE IS ONLY FOR FAMILY MEDICINE SPECIALISTS, OTHER CLINICAL SPECIALISTS, MEDICAL OFFICERS, HOUSE OFFICERS, NURSES AND ASSISTANT MEDICAL OFFICERS

	**(D) CONTINUITY OF CARE**			
18.	Among the referrals that you have received over the past one month, how frequent did you receive patient's disease-related information from the referring provider?	◯ Yes, always or usually<br>◯ Only occasionally<br>◯ Rarely or never<br>◯ Not applicable		
19.	To what extent do medical specialists feedback to you after they have finished the treatment or diagnostics of your patients?	◯ Almost always<br>◯ Usually<br>◯ Occasionally<br>◯ Seldom or never<br>◯ Not applicable		
20.	After a patient has been discharged, how long does it usually take to receive a (summary) discharge report from the hospital most frequented by your patients?	◯ 1-4 days<br>◯ 5-14 days<br>◯ 15-30 days<br>◯ More than 30 days<br>◯ I rarely or have never received a discharge report<br>◯ Not applicable		
21.	To what extent are you involved in health education as regards the following topics: (more than one answer is possible)	Not Involved	In connection with normal patients contacts (During one to one consultation)	In group sessions or special programmes
23a.	Smoking cessation	☐	☐	☐
23b.	Diet	☐	☐	☐
23c.	Physical Exercise	☐	☐	☐

	**(E) RISK STRATIFICATION**		
22.	Do you perform cardiovascular risk stratification?	◯ Yes<br>◯ No<br>◯ Don't know	If yes, please specify tool:<br>□ Framingham risk score<br>□ WHO/ISH<br>□ Pooled cohort equation<br>□ Risk factor calculation (as per Hypertension CPG 2013)<br>□ SCORE risk chart<br>□ Others, please specify: ___________

THIS QUESTIONNAIRE IS ONLY FOR FAMILY MEDICINE SPECIALISTS, OTHER CLINICAL SPECIALISTS, MEDICAL OFFICERS, HOUSE OFFICERS, NURSES AND ASSISTANT MEDICAL OFFICERS

**(F) JOB SATISFACTION**					
**To what extent do you agree with the following statements?**		**Strongly Agree**	**Agree**	**Disagree**	**Strongly Disagree**
23.	I feel that some parts of my work do not really make sense	o	o	o	o
24.	My work still interests me as much as it ever did	o	o	o	o
25.	My work is overloaded with unnecessary administrative detail	o	o	o	o
26.	I have too much stress in my current job	o	o	o	o
27.	Being a healthcare provider is a well-respected job	o	o	o	o
28.	In my work there is a good balance between effort and reward	o	o	o	o

**(I)Triage Staff** (Only for providers who select "Triage staff" in Q10)						
For the past 1 month,		**Never**	**Seldom**	**Occasionally**	**Usually**	**Always**
35.	How often is CVD screening offered to all patients ≥ 30 years old at triage counter?	o	o	o	o	o
