# Supplementary material for: The effects of enhanced primary healthcare interventions on primary care providers’ job satisfaction
Source: BMC Health Serv Res. 2020 Apr 15;20:311. doi: 10.1186/s12913-020-05183-9 (PMC7158075; doi:10.1186/s12913-020-05183-9)
Supplement: Supplementary file 3 — Additional file 3: Table S1. Changes in job satisfaction among cohort group following EnPHC Interventions. [file 12913_2020_5183_MOESM3_ESM.docx]

Supplementary Table 1 Changes in job satisfaction among cohort group following EnPHC Interventions

| Job satisfaction | Intervention Group  n = 352 | | | | | Control Group  n = 310 | | | | | Difference^*^ | Coefficient (SE) | 95% CI | *p*-value |
| --- | --- | --- | --- | --- | --- | --- | --- | --- | --- | --- | --- | --- | --- | --- |
|  |  | | |  | |  | |  | | |  |  |  |  |
| 1. Some parts of my work do not really make sense | 2.62 | (0.79) | 2.51 | | (0.77) | 2.60 | (0.76) | | 2.58 | (0.78) | -0.09 | -0.089 (0.107) | -0.299, 0.121 | 0.407 |
| 1. My work still interests me as much as it ever did | 3.10 | (0.58) | 3.30 | | (0.60) | 3.11 | (0.56) | | 3.28 | (0.61) | 0.03 | 0.030 (0.077) | -0.122, 0.181 | 0.700 |
| 1. Overloaded with unnecessary administrative detail | 2.31 | (0.67) | 2.35 | | (0.71) | 2.25 | (0.73) | | 2.42 | (0.73) | -0.13 | -0.145 (0.077) | -0.296, 0.005 | 0.058 |
| 1. Too much stress | 2.47 | (0.72) | 2.38 | | (0.72) | 2.45 | (0.71) | | 2.52 | (0.67) | -0.16 | -0.166 (0.075) | -0.313, -0.019 | **0.027** |
| 1. Well-respected job | 3.37 | (0.69) | 3.36 | | (0.73) | 3.37 | (0.65) | | 3.38 | (0.70) | 0 | -0.025 (0.080) | -0.181, 0.132 | 0.759 |
| 1. Good balance between effort and reward | 2.86 | (0.69) | 2.94 | | (0.65) | 2.93 | (0.61) | | 2.98 | (0.62) | 0.03 | 0.023 (0.080) | -0.135, 0.180 | 0.778 |

CI: confidence interval

*Change from baseline to 17 months, intervention group versus control group

Note: Outcomes are adjusted for age, gender, educational level, professional roles, working duration in primary care settings, hours spent per week on direct patient care, location of clinics (urban/rural)
