# Supplementary material for: The effects of enhanced primary healthcare interventions on primary care providers’ job satisfaction
Source: BMC Health Serv Res. 2020 Apr 15;20:311. doi: 10.1186/s12913-020-05183-9 (PMC7158075; doi:10.1186/s12913-020-05183-9)
Supplement: Supplementary file 4 — Additional file 4. [file 12913_2020_5183_MOESM4_ESM.pdf]

HEALTHCARE PROVIDER INFORMATION SHEET

1. **Title of study:** Evaluation of the Enhanced Primary Healthcare (EnPHC) initiative in public health clinics in Malaysia

2. **Institution:** Ministry of Health Malaysia

3. **Introduction:**
   You are invited to participate in a research study because you are working in one of the health clinics that have been sampled into the study. The details of the study are described in this document. It is important that you understand why the research is being done and what it will involve. Please take your time to read through and consider this information carefully before you decide if you are willing to participate. If anything is unclear or you would like more information, please ask the study staff.

   This study has been approved by the Medical Research and Ethics Committee, Ministry of Health Malaysia.

4. **What is the purpose of the study?**
   The purpose of this study is to evaluate the EnPHC initiative in public health clinics in Malaysia. We would like to get your views on the clinic services. Your views will help in planning of future health services.

5. **What will happen if I decide to take part?**
   You will be interviewed by an interviewer based on a questionnaire that has been developed for this study. The interview may take place at the end of your clinic session or at a convenient time for you.

6. **Will there be any harm related with my participation?**
   No. We only require approximately **30 minutes** of your time to complete the questionnaire.

7. **What are the benefits from my participation?**
   There are no direct benefits for your participation. However, your participation will help to enhance services provided in the public health clinics.

8. **Can I refuse to participate?**
   Yes. Your participation in this study is **voluntary**. You may withdraw from participation at any point. Should you choose to decline, we would be grateful if you could help us in completing a non-responder form, which will take approximately one minute to complete. **Your refusal to participate will not affect your work or the working condition in this clinic**.

9. **Will the data obtained from this study be kept confidential?**
   Yes. All your information obtained in this study will be kept and handled in a confidential manner, in accordance with applicable laws and/or regulations. When publishing or presenting the study results, your identity will not be revealed.

10. **Who should I call if I have questions?**
    If you have any questions about the study, please contact:

Datin Dr Sheamini Sivasampu
Healthcare Statistics Unit (HSU),
Clinical Research Centre (CRC),
Ministry of Health Malaysia
Telephone: 03 4043 9300/9400
