# Supplementary material for: The effects of enhanced primary healthcare interventions on primary care providers’ job satisfaction
Source: BMC Health Serv Res. 2020 Apr 15;20:311. doi: 10.1186/s12913-020-05183-9 (PMC7158075; doi:10.1186/s12913-020-05183-9)
Supplement: Supplementary file 5 — Additional file 5. [file 12913_2020_5183_MOESM5_ESM.pdf]

NON- RESPONSE SURVEY FORM (HEALTHCARE PROVIDER)

Name of the clinic: ____

1. Gender
   ○ Male
   ○ Female

2. What is your year of birth?
   Year of birth: 19□□

3. Number of years in practice in primary care
   Please fill in: ___________years

4. What are the reasons for your not participating in this study?
   □ Time constraints
   □ Concerned about my privacy and revealing information about myself
   □ Not interested as the outcome will not affect me
   □ I expect an incentive for participating
   □ I am afraid that the information collected will be used against me or to my disadvantage
   □ I have answered this survey before
   □ Others, please specify___________________

5. To what extent do you agree with the following statements?

		**Strongly Agree**	**Agree**	**Disagree**	**Strongly Disagree**
A	I feel that some parts of my work do not really make sense	○	○	○	○
B	My work still interests me as much as it ever did	○	○	○	○
C	My work is overloaded with unnecessary administrative detail	○	○	○	○
D	I have too much stress in my current job	○	○	○	○
E	Being a healthcare provider is a well-respected job	○	○	○	○
F	In my work, there is a good balance between effort and reward	○	○	○	○

THANK YOU FOR YOUR COOPERATION!
